# Supplementary material for: Possibilities and limitations of antisense oligonucleotide therapies for the treatment of monogenic disorders
Source: Commun Med (Lond). 2024 Jan 5;4:6. doi: 10.1038/s43856-023-00419-1 (PMC10770028; doi:10.1038/s43856-023-00419-1)
Supplement: Supplementary file 2 — Supplementary Information [file 43856_2023_419_MOESM2_ESM.pdf]

Supplementary Files

N=1 Collaborative

https://www.n1collaborative.org/who-we-are

info@n1collaborative.org

Steering Committee:

Annemieke Aartsma-Rus - Dutch Center for RNA Therapeutics, Leiden, the Netherlands

Erika Augustine - Kennedy Krieger Institute, Baltimore, United States

Scott Demarest - Children's Hospital Colorado, Denver, United States

Richard Finkel - St. Jude's Children's Hospital, Memphis, United States

Keith Flanagan - Flanagan Strategies, Washington, United States

Nina Green - Boston Children's Hospital, Boston, United States

John Keilty - Third Rock Ventures, Boston, United States

Ashley Kuniholm - Boston Children's Hospital, Boston, United States

Stefanie Leonard - N=1 Collaborative, Somerville, United States

Erin McConnell - Carleton University, Ottawa, Canada

Nicole Nolen – N=1 Collaborative, Somerville, United States

Rebecca Schüle - University of Heidelberg, Heidelberg, Germany

Matthis Synofzik - University of Tübingen, Tübingen, Germany

Bryan J. Traynor - National Institute of Aging, Bethesda, United States

Julia Vitarello - Mila's Miracle Foundation, Boulder, United States

Jonathan Watts - RNA Therapeutics Institute, UMass, Massachusetts, United States

Winston Yan - Harvard Medical School, Boston, United States

Timothy Yu - Boston Children's Hospital, Boston, United States
